# How Fungal Glycans Modulate Platelet Activation via Toll-Like Receptors Contributing to the Escape of *Candida albicans* from the Immune Response

**DOI:** 10.3390/antibiotics9070385

**Published:** 2020-07-07

**Authors:** Samir Jawhara

**Affiliations:** 1 Institut National de la Santé et de la Recherche Médicale U1285, Faculté de Médecine, 1 Place Verdun, 59000 Lille, France; samir.jawhara@univ-lille.fr; Tel.: +33-(0)3-20-62-35-46; Fax: +33-(0)3-20-62-34-16; 2 UMR 8576-UGSF-Unité de Glycobiologie Structurale et Fonctionnelle, Centre National de la Recherche Scientifique, University of Lille, 59000 Lille, France

**Keywords:** platelets, *Candida albicans*, glycans, β-glucans, chitin, Toll-like receptors, thrombus, aggregation

## Abstract

Platelets are essential for vascular repair and for the maintenance of blood homeostasis. They contribute to the immune defence of the host against many infections caused by bacteria, viruses and fungi. Following infection, platelet function is modified, and these cells form aggregates with microorganisms leading, to a decrease in the level of circulating platelets. During candidaemia, mannans, β-glucans and chitin, exposed on the cell wall of *Candida albicans*, an opportunistic pathogenic yeast of humans, play an important role in modulation of the host response. These fungal polysaccharides are released into the circulation during infection and their detection allows the early diagnosis of invasive fungal infections. However, their role in the modulation of the immune response and, in particular, that of platelets, is not well understood. The structure and solubility of glycans play an important role in the orientation of the immune response of the host. This short review focuses on the effect of fungal β-glucans and chitin on platelet activation and how these glycans modulate platelet activity via Toll-like receptors, contributing to the escape of *C. albicans* from the immune response.

Platelets, also known as thrombocytes, are the smallest anuclear cells circulating in the blood, and originate from fragmentation of the cytoplasm of large cells, known as megakaryocytes, in the bone marrow [1,2]. Platelets have a glycoprotein-rich membrane, which is involved in platelet function, and also receptors involved in platelet adhesion, activation, aggregation and inhibition such as integrins [1,2]. Among these integrins, integrin αIIβ1 plays an important role in the adhesion process to collagen and to the injured vessel wall [3]. Integrin αIIbβ3 is the dominant integrin on the platelet surface and plays an essential role in the platelet response, as its activation is essential for platelet–platelet interactions and thrombus formation [4]. Integrin αIIbβ3 mediates platelet aggregation through the binding of plasma fibrinogen [4]. Other surface receptors are present on platelets and are involved in the immune defence, such as lectin-type oxidized LDL receptor 1 (LOX-1) and P-selectin. The latter is expressed only on activated platelets and supports the secretion and immobilisation of platelet chemokines on the activated endothelium and also on leukocyte PSGL-1 (P-selectin glycoprotein ligand 1) increasing the circulation and recruitment of leukocytes on the endothelium. Platelet–neutrophil complexes have been shown to be markers of platelet activation. Additionally, this interaction between platelets and neutrophils is observed in a wide range of inflammatory conditions [5,6]. Platelets interact with neutrophils during infection, inflammation and thrombosis, and modulate each other’s functions via different interactions including platelet P-selectin binding to neutrophil PSGL-1 and platelet glycoprotein Ibα binding to neutrophil integrin αMβ2 (CD11b/CD18) [7,8]. Activated platelets can initiate or boost different neutrophil responses including phagocytosis and the production of oxygen radicals and neutrophil extracellular trap (NET). With regard to platelet and monocyte interactions, Wrigley et al. showed that cross-talk between monocytes and platelets is reflected by the formation of monocyte–platelet aggregates in patients with ischemic heart failure [9]. Additionally, platelet factor 4 (PF-4) is a potent activator of phagocytosis and reactive oxygen radical formation in human monocytes [10].

Platelets possess the purinergic receptors P2Y1, P2Y12 and P2X1 [3,4]. Receptor P2X1 is associated with a calcium channel and provokes, in response to ATP, the penetration of calcium into the inside of the platelet, contributing to a change in platelet shape, pseudopodia formation, platelet degranulation and platelet activation [11]. Activation of the P2X1 receptor by ATP alone does not induce platelet aggregation, whereas platelet aggregation induced by thrombin through protease-activated receptor 1 (PAR1) is enhanced by activation of the P2X1 receptor [12]. P2Y12 and P2Y1 receptors are G-protein-coupled receptors activated by ADP. P2Y1 receptor activation by ADP results in a change in platelet shape and reversible aggregation while activation of P2Y12 leads to sustained platelet aggregation but without a change in platelet shape [13,14].

The platelet cytoplasm is composed of a large number of organelles including mitochondria, glycogen grains and different types of granules whose contents are released during activation. Among the different types of granules, α-granules are the most abundant. These contain a large number of proteins that are specific to platelets, such as chemokines like PF-4, coagulation factors (V, XI, XIII), β-thromboglobin, plasma IgGs, growth factors (PDGF, TGF-β) and adhesion proteins such as von Willebrand factor (vWF), and fibrinogen.

Dense granules mainly contain nucleotides such as ATP and ADP, serotonin and Ca2^+^ (Figure 1) [15]. These platelets are important in vascular repair and the maintenance of haemostasis, involving the maintenance of a constant volume of blood in the vessels (Figure 1). During a lesion to the endothelium, they are the first to arrive and adhere rapidly to proteins in the sub-endothelial matrix such as collagen and vWF, using glycoprotein complexes (GPIb-V-IX and GPVI) present in the membrane. This adhesion provokes platelet activation, principally by the engagement of Ca^+^ flow inducing the secretion of thromboxane A2 (TxA2), ADP, fibrinogen and other factors that will allow platelet recruitment, but also platelet aggregation via integrin αIIbβ3. This aggregation results in the formation of a clot, also called a white thrombus, helping to close the vascular breach (Figure 1) [1].

In addition to the role of platelets in haemostasis, they rapidly move to the site of a lesion and thus a potential infection. Platelets are the first immune cells to participate in the host defence against many bacterial, viral and fungal infections (Figure 1). Platelets express Toll-like receptors (TLRs), which are the key receptors in the interaction between innate and adaptive immunity, and their expression plays a role in the modulation of platelet secretion [16]. Among the TLRs identified on platelets, Shiraki et al. showed that TLR1 and TLR6 are expressed at mRNA and protein levels in human platelets [17]. Additionally, other studies have demonstrated that TLR2 and TLR4 expression was located not only on the surface, but also in the cytoplasm of platelets [18,19]. Platelet TLR7, which localizes to the endosomal compartment, has been shown to play an important role in the recognition of ssRNA viruses during viral infection [20]. In terms of platelet TLR9, it is expressed on the surface and in the cytoplasm of inactivated platelets, and the activation of platelets with thrombin increases TLR9 surface expression [21]. Platelets also use functional signalling pathways such as protein MyD88, which lead to the activation of nuclear activation factor NF-κB [22]. With regard to the role of platelet TLRs in haemostasis and pathogen infection, TLR2 in association with TLR1 or TLR6 recognizes bacterial lipoproteins and is involved in the formation of thrombi [23]. Different investigators have reported that TLR2 is involved in platelet aggregation, adherence to collagen and the formation of platelet–neutrophil aggregates [23,24]. Blair et al. showed that the stimulation of TLR2 by bacterial components in human platelets induces a thrombo-inflammatory response through the activation of phosphoinositide 3-kinase [23]. Additionally, the activation of TLR2-induced platelet aggregation and secretion depends on P2X1-mediated calcium mobilization, the production of TxA2 and ADP receptor activation. TLR4 stimulation by lipopolysaccharides (LPS) induces platelet secretion and potentiates platelet aggregation via TLR4/MyD88 and the cGMP-dependent protein kinase pathway [24]. The release of histones from dying cells is associated with microvascular thrombosis, while blocking platelet TLR2 and TLR4 decreases the percentage of activated platelets and reduces the amount of thrombin generated, indicating that TLR2 and TLR4 mediate the activation process [25]. During sepsis, platelet TLR4 activation induces platelet binding to adherent neutrophils, leading to robust neutrophil activation and NET formation in liver sinusoids and pulmonary capillaries, which facilitate bacterial capture [26]. Wang et al. showed that an increase in platelet TLR4 expression along with platelet activation in patients with sepsis is closely related to the incidence of thrombocytopenia [27]. TLR2 and TLR7 stimulation can trigger platelet degranulation and play an important role in procoagulant production and mediating sepsis-induced coagulopathy [28]. Platelet TLR7 stimulation promotes the translocation of P-selectin to the cell surface and a consequent increase in platelet–neutrophil adhesion [20]. However, the stimulation of platelet TLR9 by endogenous ligands induces the phosphorylation of IRAK1 and AKT, promoting platelet hyperreactivity and thrombosis [29].

During the course of a microbial infection, platelets can be activated by three mechanisms [30,31]. The first involves an inflammatory response against the pathogen, leading to the secretion of proinflammatory cytokines by leukocytes, resulting in platelet activation. The second involves bacteria, which may secrete molecules that activate platelets. Finally, other microorganisms can adhere directly to platelets and activate them, particularly Staphylococci and fungi [31,32].

However, pathogens do not all interact in the same way with platelets, which may or may not be activated in response to these stimuli. It is interesting to note that, in the presence of some strains of bacteria such as *Staphylococcus epidermidis* and *Streptococcus pneumoniae*, platelets are only weakly activated and aggregate non-irreversibly, and an infection is often linked to thrombocytopenia, which may be a sign of increased severity [33]. Of note, TLR-mediated platelet responses to different bacterial species vary. Berthet et al. showed that platelets can discriminate between *Escherichia coli* and *Salmonella* species via TLR4 signalling and differential cytokine secretion, and modulate the innate immune response appropriately for pathogens exhibiting different types of ‘danger’ signals [34].

Clinical and experimental studies have demonstrated a reduction in the number of platelets, below the normal platelet threshold (150 × 10^3^ platelets/µL), during infection with *Candida albicans* [35,36]. Holder et al. demonstrated that mice infected with *C. albicans* showed a significant reduction in circulating platelets and a shortening of clotting time within hours after challenge [36]. In line with this study, Robert et al. showed that, during *C. albicans* infection, platelets lose their discoid shape and produce pseudopods, suggesting that fibrinogen receptors are likely present on *C. albicans* and are involved in a bridge between *C. albicans* and activated platelets [37,38]. Other studies have shown that the deposition of platelets and fibrin on a lesion of endocarditis supports the installation of microorganisms like *C. albicans* leading to infectious endocarditis, and that fungal glycan fractions could play a role in the adherence of *C. albicans* to this fibrin–platelet matrix, thereby activating platelets, supporting their aggregation and provoking thrombotic endocarditis [39,40].

*C. albicans* is a commensal yeast and a natural saprophyte of the digestive tract and vaginal mucosa of humans [41]. Excessive fungal colonisation of the digestive mucosa is associated with risk factors such as immunosuppression and changes to the digestive mucosa support translocation of the yeast across the digestive epithelial barrier and haematogenous dissemination, leading to severe disseminated infections [42].

*C. albicans* possesses several virulence molecules including proteases, phospholipases, lipases and esterases that help the fungal cells to escape from the host immune responses. *C. albicans* lipases facilitate active penetration of the yeast into the host cells and are involved in *C. albicans* invasion of tissues by hydrolysing the lipid components of the host cell membranes [43]. Similarly, *C. albicans* aspartic proteases (Saps) contribute to the degradation of host cell components, including cells of the innate immune system [44]. Sap1–10 are involved in the adhesion of *C. albicans* to host cells and the invasion of tissues through the degradation of cell surface structures [45]. Gropp et al. showed that *C. albicans* evades human complement attack by the secretion of Saps, indicating that this fungal pathogen has developed this enzymatic strategy to escape the host immune defence [46]. Additionally, integrin αMβ2 and αXβ2 receptors are inactivated by *C. albicans* Sap2 which may represent an additional way for *C. albicans* to evade the host innate immune system [47]. Interestingly, the hyphal form of *C. albicans* secretes cytolytic peptide toxin, named ‘Candidalysin’ which contributes to epithelial membrane damage and triggers a danger response signalling pathway promoting *C. albicans* infection [48]. A further strategy of *C. albicans* invasion involves the soluble ligand Pra1p (pH-regulated antigen 1 protein) [49,50,51]. Soloviev et al. showed that *C. albicans* releases a soluble ligand Pra1p which is recognized by leukocyte αMβ2 [49]. This Pra1-integrin αMβ2 complex blocks leukocyte adhesion to *C. albicans*, an interaction which is critical in the killing of the fungus, indicating that soluble Pra1p may assist the fungus in escaping host surveillance [49].

The cell wall of *C. albicans* is composed of 80-90% polysaccharides associated with proteins and lipids. The external layer is composed mainly of phosphopeptidomannan (PPM) and phospholipomannan (PLM) and the internal layer contains β-1,3 and β-1,6 glucans representing 40% and 20% of the dry weight, respectively, as well as chitin, which only represents 2% of dry weight under physiological conditions [42]. Chitin, a β(1,4)-linked homopolymer of N-acetylglucosamine, is covalently attached to β-glucans in the *C. albicans* cell wall. The cell wall is a dynamic structure which undergoes constant modifications [41]. In its hyphal form, *C. albicans* contains three to four times more chitin, localized mainly on the surface on yeast scars formed after budding. This internal layer of the *C. albicans* cell wall is surrounded by superficial mannans, which are the first target of the immune response, resulting in mannan–immune receptor interactions. However, the unmasking of β-glucan and chitin by exposure to host enzymes, acidic environments or antifungal drugs has major immunomodulatory consequences. Sherrington et al. showed that the unmasking of underlying immune-stimulatory β-glucan in acidic environments enhanced innate immune recognition of *C. albicans* by macrophages and neutrophils and induced a stronger proinflammatory cytokine response, driven through the C-type lectin-like receptor [52]. β-glucans and chitin synthesis are an attractive target for antifungal therapies and combinations of β-glucan and chitin synthase inhibitors are more potent against *C. albicans* than individual drug treatments [53]. All of these glycans exposed on the cell wall of *C. albicans* are recognized by pattern recognition receptors (PRRs) expressed on the surface of host cells and are able to modulate the immune response [26]. β-glucan is recognized by different receptors such as dectin-1, αMβ2 and TLR2 that are expressed in host cells, including neutrophils, macrophages, monocytes, dendritic cells and NK cells. β-glucans can interact with dectin-1, in association with galectin-3 or TLR2, promoting the modulation of signalling pathways and an increase in the proinflammatory cytokine response [54,55,56]. The molecular structure of β-glucans plays an important role in their immunological properties, including polymer length and the degree of branching, their solubility and their influence on the activation or inhibition of leukocyte receptors. It has been shown that soluble β-glucans derived from yeasts could block receptors such as dectin-1 and αMβ2 and prevent multivalent binding necessary for a strong triggering of leukocyte inflammatory responses [51,55]. In line with this observation, the oral administration of soluble β-glucans derived from *Candida* to mice decreased the overgrowth of aerobic bacteria, in particular *E. coli* and *Enterococcus faecalis* populations, and overgrowth of *Candida*, as well as the production of inflammatory parameters [57]. Additionally, β-glucan treatment increased IL-10 production via PPARγ sensing, promoting the attenuation of inflammation in mice [57].

*C. albicans* can switch between yeast and filamentous morphologies, which are crucial to *C. albicans* pathogenicity. Gantner et al. showed that β-glucan from blastoconidia is not recognized by dectin-1, but unmasking of β-glucan during the process of budding promotes the activation of dectin-1 and triggers antifungal inflammatory responses in macrophages while *C. albicans* filaments which do not expose β-glucan in bud scars failed to activate dectin-1-mediated defences [58]. Additionally, the recognition of *C. albicans* by TLR is dependent on the shape of the yeast. *C. albicans* yeast forms can activate both TLR2 and TLR4 in peripheral blood mononuclear cells and peritoneal macrophages while the hyphal form is not recognized by TLR4, the receptor which induces proinflammatory cytokine production, including IFN-γ [59].

Among the receptors that recognize chitin are fibrinogen C domain-containing protein 1 (FIBCD1) and NOD 2 [60,61]. FIBCD1 binds calcium to chitin via a conserved S1 binding site and promotes endocytosis [60]. Rivera et al. showed that FIBCD1 is expressed in epithelial cells of human lung that recognize the fungal cell wall. The sensing of the fungal cell wall by FIBCD1 results in the suppression of epithelial inflammatory signalling, such as IL-8 and mucin production, indicating that FIBCD1 activation has an impact on the modulation of the immune response by decreasing antifungal elimination through the disruption of neutrophil recruitment and mucin production [62]. Wagner et al. showed that chitin from *C. albicans* increases arginase-1 activity in human macrophages and suppresses NO synthesis, suggesting that chitin can influence macrophage function by reducing antimicrobial activities and mediating fungal survival [63]. In one study, chitin derived from *C. albicans* blocked *C. albicans* recognition by human peripheral blood mononuclear cells (PBMCs) and murine macrophages, leading to a significant reduction in production of cytokines indicating that chitin is capable of influencing immune recognition by blocking dectin-1-mediated engagement with fungal cell walls [64]. Addition investigations have shown that the *Candida* cell wall undergoes various changes during its passage through the digestive tract and demonstrated a significant increase in chitin, which is involved in promoting persistence of Candida in the gut [65].

Clinically, these glycans are released into the circulation during infection and their detection allows the early diagnosis of invasive fungal infections [66]. Furthermore, β-glucans and mannans are released into the circulation during an invasive fungal infection and can be detected, on average, 10 days before the appearance of the first clinical signs [67]. Sims et al. demonstrated that during an invasive fungal infection, the level of circulating β-glucans decreased significantly in patients who were successfully treated and increased excessively in cases of therapeutic failure, and concluded that the detection of circulating β-glucans is a key tool for the therapeutic monitoring of invasive candidosis [68]. These data demonstrate a close link between β-glucans and fungal infection, but the role of the immune defence system, and platelets in particular, in this process has not been widely studied.

During the course of infection, platelets interact directly with leukocytes [69,70]. The expression of P-selectin as well as activation of integrin αIIbβ3 facilitates the adherence of platelets to neutrophils and other leukocytes [69,70]. This interaction induces the recruitment of neutrophils in infected tissues. Platelets also increase the microbicidal activity of neutrophils by increasing the level of TNFα [71] and by secreting IL-1β and TxA2, which stimulate the oxidative metabolism of neutrophils [72]. Vancraeyneste et al. showed that the addition of *C. albicans* β-glucans at a low concentration of 2 µmol/L in plasma reduces thrombin production and, as a consequence, β-glucan fractions have activity that is similar to that of low molecular weight heparin [73]. In contrast, the progressive increase in β-glucan concentration in plasma decreases coagulation until these β-glucan fractions no longer affect thrombin production. This indicates that β1,3-glucan fractions have a dose-dependent effect [73].

Under physiological conditions, low doses of thrombin induced the activation of the PAR-1 receptor, which led to the activation of G proteins coupled to PAR-1 [74]. This process is helped by the release of ADP/ATP and TxA2 which bind to their respective receptors on the platelet surface, allowing platelet self-activation as well as maintenance of the active state [75]. Diglucoside (Glc2) and pentaglucoside (Glc5) fractions derived from *C. albicans* had anti-thrombotic activity and reduced platelet activation and aggregation [73]. This activity was due to the direct binding of glycan factions to platelets. In addition, Glc2 and Glc5 reduced the expression of receptors such as P-selectin and activation of integrin αIIbβ3 [73]. These data were confirmed by measuring ATP and platelet proteins released by platelets activated by thrombin. These observations suggest that β-glucans have an anti-aggregant effect on platelets [73]. This effect varies according to the size and structure of the fraction studied [76]. It has been demonstrated that native β1,3-glucans (of high molecular weight) derived from *S. cerevisiae* possess platelet anti-aggregant and anti-oxidant properties [77]. These polysaccharides reduce the activity of the enzyme cyclo-oxygenase in the arachidonic acid cascade and reduce the production of superoxide radicals in platelets by stimulated biological agonists [76]. Istabishi et al. demonstrated that soluble fractions inhibit TNFα production by macrophages compared to insoluble β-glucans [78]. β-glucan fractions exert an effect on the processes of platelet adhesion. Glc2 and Gc5 fractions reduced the adhesion of platelets to fibrinogen, *C. albicans* hyphae and neutrophils supporting the observation that glucosidic fractions modulate the self-activation of platelets by promoting a process by which *C. albicans* can escape from the immune response [73].

TLRs are involved in the recognition of *C. albicans* cell wall glycans, including β-glucans, allowing platelets to participate in the host defence against many infections [79]. The level of expression of TLR2 and TLR4 mRNA in platelets pre-treated with β1,3-glucans was closely correlated with the level of expression of TLR4 and TLR2 proteins [73]. Furthermore, pre-treatment of platelets with β1,3-glucan fractions did not modulate the level of expression of TLR2 while it reduced platelet activation mediated by TLR4 by increasing the production of TGF-β1 and release of ATP. Blocking this receptor with an anti-TLR4 antibody abolished the effect of β1,3-glucans on platelets indicating that the effects of β1,3-glucans on platelets are mediated by TLR4 [73]. Chai et al. demonstrated that β-glucan modulation of host defence mechanisms mediated via TLR4 by a reduction in proinflammatory cytokine production was a strategy of immune evasion by *Aspergillus fumigatus* [80]. Furthermore, β-glucan was able to suppress endotoxin mediated via TLR4 by inducing the release of proinflammatory cytokines and by affecting the NF-kB pathway [81,82]. In microglial cells, β-glucans also reduced the production of cytokines, mediated not only by TLR4 but also by TLR2 [82].

Like β-glucans, chitin is also released into the circulation during fungal infection and is able to modulate the immune response [83]. A murine model of inflammatory colitis confirmed the role of fungal chitin in anti-inflammatory responses [65].

The oral administration of chitin derived from *Candida* significantly decreased inflammation of the colon and had a significant effect on the elimination of fungi from the digestive tract [65]. This decrease was associated with the expression of TLR8 and NOD2 mRNA in colonic cells [65]. However, to our knowledge, no receptor for fungal chitin has been described on platelets to date. Wagener et al. identified NOD2, TLR9 and the mannose receptor as potential receptors for chitin in a macrophage model [61]. Leroy et al. showed that chitin from *C. albicans* decreased platelet adherence to neutrophils and hyphae of *C. albicans*, suggesting a process by which *C. albicans* can escape from cells of innate immunity by releasing cell wall polysaccharides into the close environment or blood circulation [84]. Chitin also reduced the aggregation and activation of integrin αIIbβ3, supporting the idea that chitin has anti-inflammatory properties. In contrast to TLR2, TLR4 and TLR9, pre-treatment of platelets with fungal chitin reduced platelet activation mediated by TLR8 stimulation by decreasing the level of intracellular calcium and the expression of P-selectin [84]. In addition, TLR8 mRNA transcript and protein expression levels increased in thrombin-activated platelets in a chitin concentration-dependent manner, supporting the idea that *C. albicans* chitin modulates platelet activation mediated by TLR8 in thrombin-activated platelets [84].

## Conclusions

We have described the role of fungal glycans in the modulation of platelet activity. These fungal glycans decrease platelet activation and modulate the production of inflammatory mediators, mediated by TLR4 or TLR8. These glycans also decrease platelet adherence to *C. albicans* and to polynuclear neutrophils, suggesting a process by which *C. albicans* can escape from cells of innate immunity by releasing cell wall polysaccharides into the close environment or the blood circulation. Finally, interaction between different *C. albicans* cell wall glycans and platelet receptors is critical in the orientation of the immune response of the host and in the pathogenesis of the yeast and infection.

It would be important to assess the virulence of clinical strains of *C. albicans* isolated from candidaemic patients with thrombocytopenia or *C. albicans* strains deficient in β-glucans or chitin through their ability to induce platelet aggregation, the secretion of proinflammatory cytokines and arterial thrombus formation in an experimental model in mice deficient in TLR. The results of these investigations may provide additional information on the role of fungal glycans in modulation of the immune response and how *C. albicans* can escape from the innate immune response. Additionally, future studies are necessary to explore the complex cross-communication of platelet TLRs and platelet C-type lectin receptors including LOX-1 in response to the pre-treatment of platelets with fungal glycans, in order to highlight new targets involved in the pathogenicity of *C. albicans* and in the modulation of platelet activation.

## Figures and Tables

**Figure 1:**
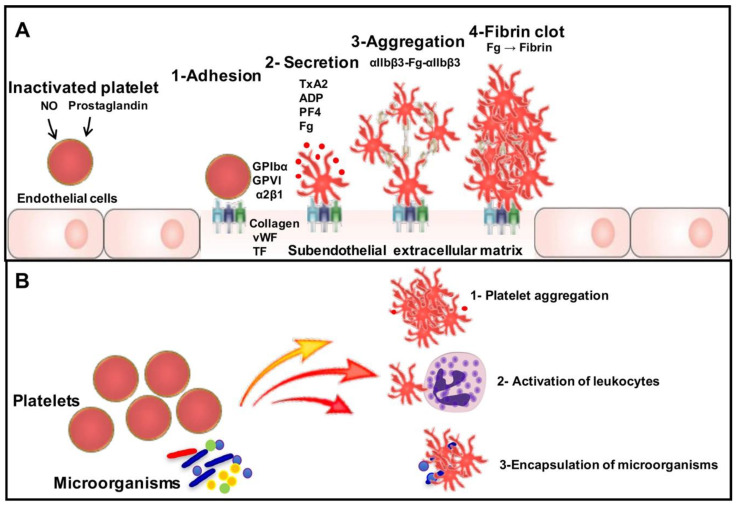
Schematic representation of platelet role in haemostasis, and during the course of infection. (**A**) Platelets circulate in an inactive state, maintained by prostacyclin (PGI2) and nitric oxide (NO) secreted by endothelial cells. During a lesion to the endothelium, collagen, vWF and tissue factor (TF) are exposed, activating platelets. These platelets adhere to the site of damage through a series of molecular interactions between platelet receptors (GPIbα, GPVI, α2β1) that bind directly to collagen and vWF, and once activated, the platelets secrete different molecules such as ADP, PF4, serotonin, fibrinogen (Fg) or TxA2, which attract circulating platelets and activate them. Inside-out signalling leads to the activation of talin and kindlin resulting in conformational changes to integrin αIIbβ3 that promote exposure of the Fg binding site and platelet aggregation via αIIbβ3-fibrinogen-αIIbβ3 bridges. Blood clotting is catalysed by thrombin, which promotes Fg binding to αIIbβ3 and converts Fg to fibrin. (**B**) During the course of infection with microorganisms, platelets can be activated by three mechanisms. The first mechanism is platelet aggregation, the second is leukocyte activation, in particular neutrophils, and the third is the encapsulation of microorganisms by platelets.
